# Could de-stressing the brain be the solution for long-term weight loss?

**DOI:** 10.15698/cst2019.02.174

**Published:** 2019-01-25

**Authors:** Florian Seyfried, Mohammed K. Hankir

**Affiliations:** 1 Department of General, Visceral, Vascular and Pediatric Surgery, University Hospital Wuerzburg, Wuerzburg, 97080 Bavaria, Germany.; 2 Department of Experimental Surgery, University Hospital Wuerzburg, Wuerzburg, 97080 Bavaria, Germany.

**Keywords:** endoplasmic reticulum stress, gliosis, hypothalamus, inflammation, leptin resistance, obesity, vertical sleeve gastrectomy

## Abstract

The obese brain is stressed and inflamed. This is mainly at the level of neurons and glial cells in the hypothalamus: a brain region where the adipokine leptin acts to control feeding and body weight. Relieving hypothalamic neuronal endoplasmic reticulum (ER) stress with the natural small molecule drugs celastrol or withaferin-A reverses the leptin resistance commensurate with obesity, producing a degree of weight loss found only with bariatric surgery. Here, recent evidence from rodent models of vertical sleeve gastrectomy (VSG) is brought to the fore which suggests that this particular bariatric surgical procedure may work in a similar fashion to celastrol and withaferin-A alongside remedying hypothalamic inflammation and gliosis. Thus, restoring and preserving healthy hypothalamic neuronal and glial cell function, be it by pharmacological or surgical means, ensures a negative energy balance in an environment constructed to promote a one - possibly through re-establishing communication between adipose tissue and the brain.

## INTRODUCTION

One just needs to take a look at a typical vending machine (often found in hospitals) to appreciate what has happened to our food and drink. The relatively sudden widespread ease of access to energy-dense meals, combined with a generally less active way of life and superimposed on a susceptible genetic background has fueled a steep rise in global obesity prevalence. This in turn has directly contributed to the increased incidence of chronic debilitating conditions such as type II diabetes, atherosclerosis, cardiovascular disease and certain cancers which are all major causes of premature death.

## ADIPOSE TISSUE AND INTESTINAL INFLAMMATION AS A CAUSE OF INSULIN RESISTANCE

**Figure 1:**
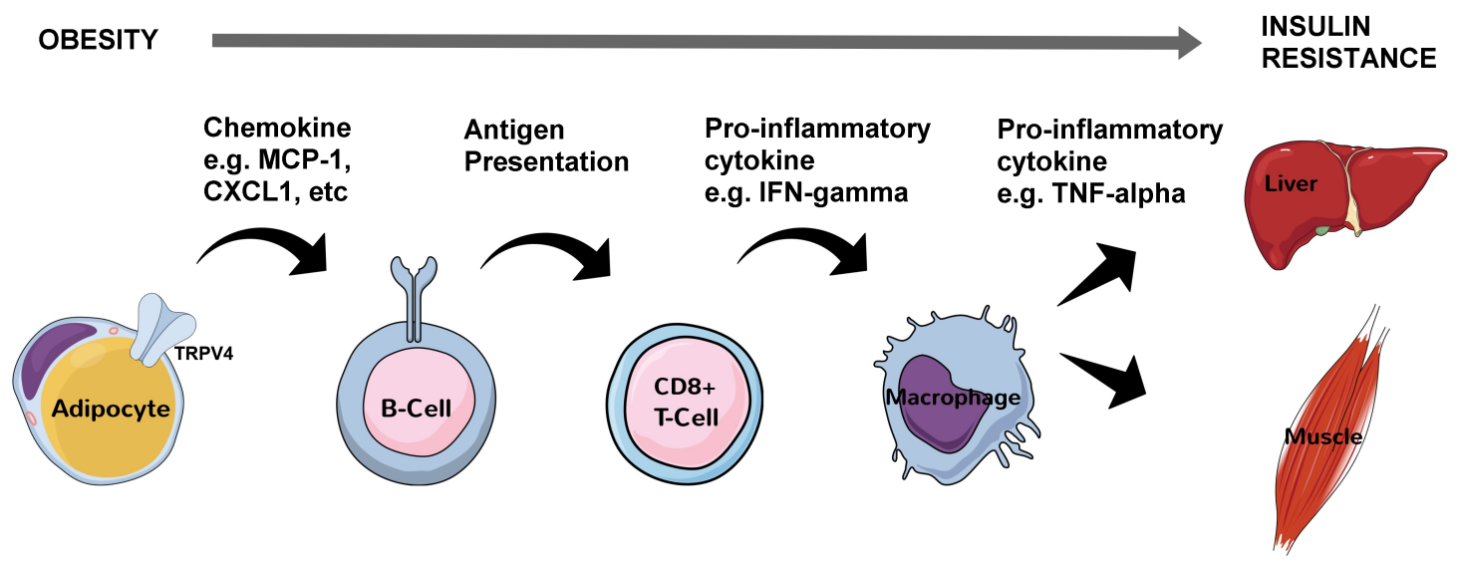
FIGURE 1: Molecular and cellular processes linking obesity with systemic insulin resistance. Swelling (hyperplasic) visceral adipocytes in obesity have hyperactivated TRPV4 which leads to changes in gene expression (through extracellular related kinase 1/2 signaling) including an increase in chemokine production. This sets into motion a sequence of molecular and cellular events ultimately leading to insulin resistance in hepatocytes and myocytes in part through inhibitory serine 120 and serine 210 phosphorylation of insulin receptor substrate 1 in these cells by TNF-α. CXCL1 - chemokine (C-X-C motif) ligand 1, IFN-gamma – interferon gamma, MCP-1 - monocyte chemoattractant protein 1, TNFα – tumor necrosis factor alpha, TRPV4 - transient receptor potential vanilloid 4.

Early efforts to disentangle the close relationship between obesity and type II diabetes focused on the pro-inflammatory cytokine tumor necrosis factor alpha (TNF-α) in visceral white adipose tissue (vWAT) [1]. A pronounced increase was found in various rodent models of obesity [1] and this marked the beginning of a powerful narrative in which increased visceral adiposity causes a state of chronic, low-grade systemic inflammation involving both the adaptive and innate immune systems. Subsequent rodent studies revealed in reverse chronological order that during obesity progression, antigen-presenting and IgG-releasing B cells are the first to arrive in vWAT, possibly attracted by chemokines released from swelling adipocytes sensed by the mechanoreceptor transient receptor potential vanilloid 4 (TRPV4) [2], followed by the recruitment of cytotoxic CD8+ T cells and then by M2 macrophages [3, 4, 5]. These white blood cells all release a plethora of pro-inflammatory cytokines including macrophage-derived TNF-α [6] that interfere with insulin receptor signaling both locally in vWAT and distally in peripheral tissues such as the liver and skeletal muscle [7] (**Figure 1**).

It is now increasingly recognized that a complex immune response also takes hold in the gut from chronic consumption of a high-fat diet due to shifts in resident microbiota species. This engenders the downregulation of regulatory T cells (Treg) and innate lymphoid cells (ILCs), which normally secrete the anti-inflammatory/intestinal barrier-protective cytokines interleukin 10 (IL-10) and IL-22, respectively, with the concomitant upregulation of cytotoxic CD8+ and Th1 T cells, which secrete the pro-inflammatory and intestinal barrier-disrupting cytokine interferon gamma (IFN-γ) [8]. The loss of gut barrier integrity itself in obesity results in systemic endotoxemia which too contributes to vWAT macrophage activation, possibly through bacterially-derived lipopolysaccharide (LPS) acting on local toll-like receptor 4 (TLR4) [8, 9]. Thus, we now know a great deal about the molecular and cellular under-pinnings of obesity-induced insulin resistance, although this has yet to be translated into an effective immune-based therapy for type II diabetes in humans.

## HYPOTHALAMIC INFLAMMATION AS A CAUSE OF LEPTIN RESISTANCE

**Figure 2:**
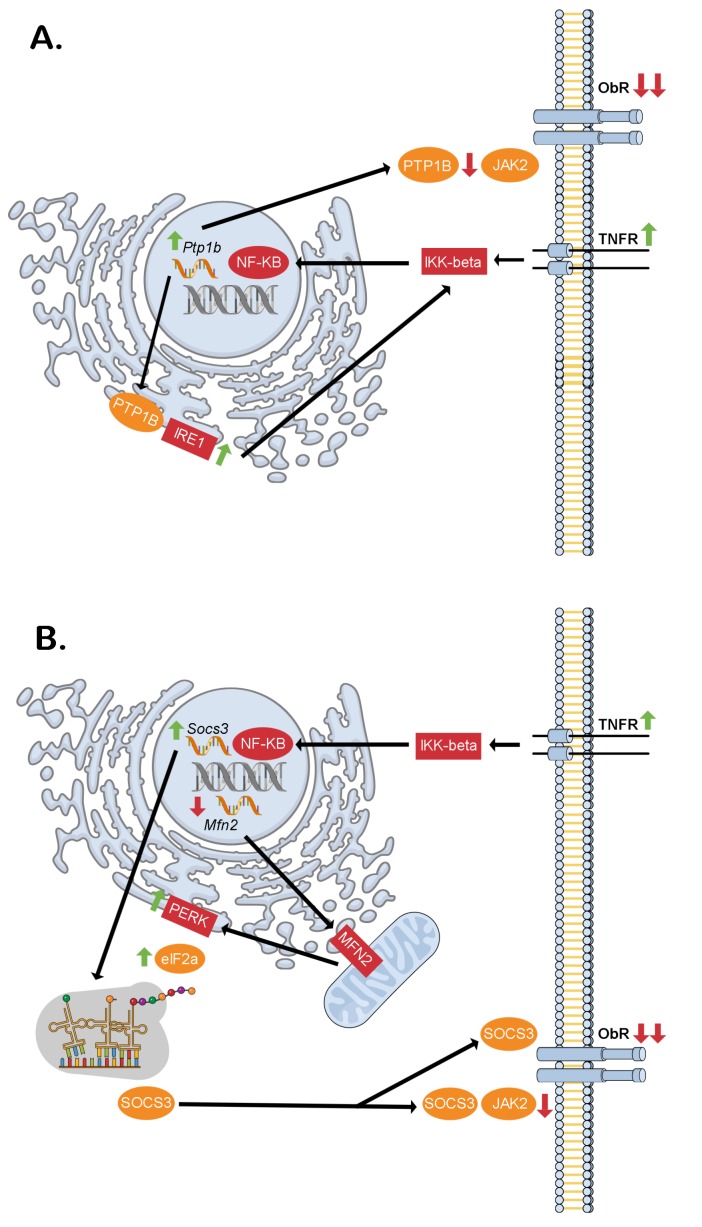
FIGURE 2: Proposed intracellular signaling cascades liking inflammation, ER stress and hypothalamic neuronal leptin resistance in obesity. **(A)** Through the dual phosphatase action of PTP1B at the ER (stimulatory on IRE1) and cell membrane (inhibitory on JAK2) downstream of TNF-α receptor activation, hypothalamic neuronal leptin receptor signaling may be blunted contributing to increased food intake and obesity. **(B)** Similarly, through the dual action of NF-KB of increasing *Socs3* transcription and decreasing *Mfn2* transcription, hypothalamic neuronal leptin receptor signaling may be blunted contributing to increased food intake and obesity. This would be through decreased mitochondrial MFN2 leading to reduced ER-mitochondrial contacts thereby causing ER stress. The PERK-eIF2α arm of this response mediates alternative translation of *Socs3* mRNA of a more stable SOCS3 variant, which lacks an amino terminus tail containing a lysine residue that is normally ubiquitinated sending the full-length SOCS3 to the proteasome for degradation. eIF2a - elongation initiation factor 2 alpha, IKK-beta - inhibitor of kappa beta kinase beta, IRE1 - inositol requiring enzyme 1, JAK2 - janus kinase 2, Mfn2 - Mitofusin-2, NK-κB - necrosis factor kappa beta, ObR – leptin receptor, PERK - protein kinase R (PKR)-like ER kinase, PTP1B - protein tyrosine phosphatase 1B, Socs3 - suppressor of cytokine signaling 3, TNFR - tumor necrosis factor receptor.

At about the same time that inflammation in the vWAT of severely obese and diabetic *ob/ob* mice was discovered [1], the *ob* gene encoding the adipokine leptin was itself cloned [10]. This generated considerable excitement about the prospects of a new and more effective obesity pharmacotherapy. However, it was soon realized that individuals with obesity are refractory to the appetite suppressing and weight lowering effects of exogenous leptin treatment [11]. Analogous to insulin resistance, inflammatory processes in the brain, specifically in hypothalamic neurons, would provide a link between obesity and the relatively newly coined term leptin resistance [12]. Furthermore, hypothalamic neuronal endoplasmic reticulum (ER) stress was also shown to develop upon chronic high-fat food consumption in mice, being both a cause and a consequence of pro-inflammatory inhibitor of kappa beta kinase beta (IKK-β) signaling [12]. That acute brain overload of the saturated fatty acid oleate was sufficient to increase hypothalamic necrosis factor kappa beta (NF-Кβ) transcriptional activity supported the concept that high-fat feeding first promotes hypothalamic neuronal inflammation and ER stress, followed by leptin resistance. Consequently, the rise in circulating leptin levels as fat mass increases fails to act as a negative feedback signal to maintain a stable body weight. Contrary to what might be expected however, ER stress does not affect leptin receptor folding in the ER and trafficking to the plasma membrane [13]. While a unifying mechanism for hypothalamic inflammation, ER stress and diminished leptin receptor signaling in obesity is still missing, the increased expression of protein tyrosine phosphatase 1B (PTP1B) caused by NF-Кβ is a likely candidate [14]. This is because beyond the established inhibitory role of PTP1B in dephosphorylating the leptin receptor effector protein janus kinase 2 (JAK2) at the cell membrane [15], it also potentiates the inositol requiring enzyme 1 (IRE1) arm of the ER stress response through its phosphatase activity at the ER [16] (**Figure 2A**). Additionally/alternatively, NF-Кβ could increase suppressor of cytokine signaling 3 (SOCS3) [12] and decrease mitofusion 2 [17] expression to directly interfere with leptin receptor signaling [18] and cause hypothalamic ER stress [19], respectively. Also, through the protein kinase R (PKR)-like ER kinase (PERK) and eukaryotic elongation initiation factor 2 alpha (eIF2-α) arm of the ER stress response, a more stable SOCS3 isoform is produced by alternative translation which would serve to further exacerbate leptin resistance [20] (**Figure 2B**).

**Figure 3:**
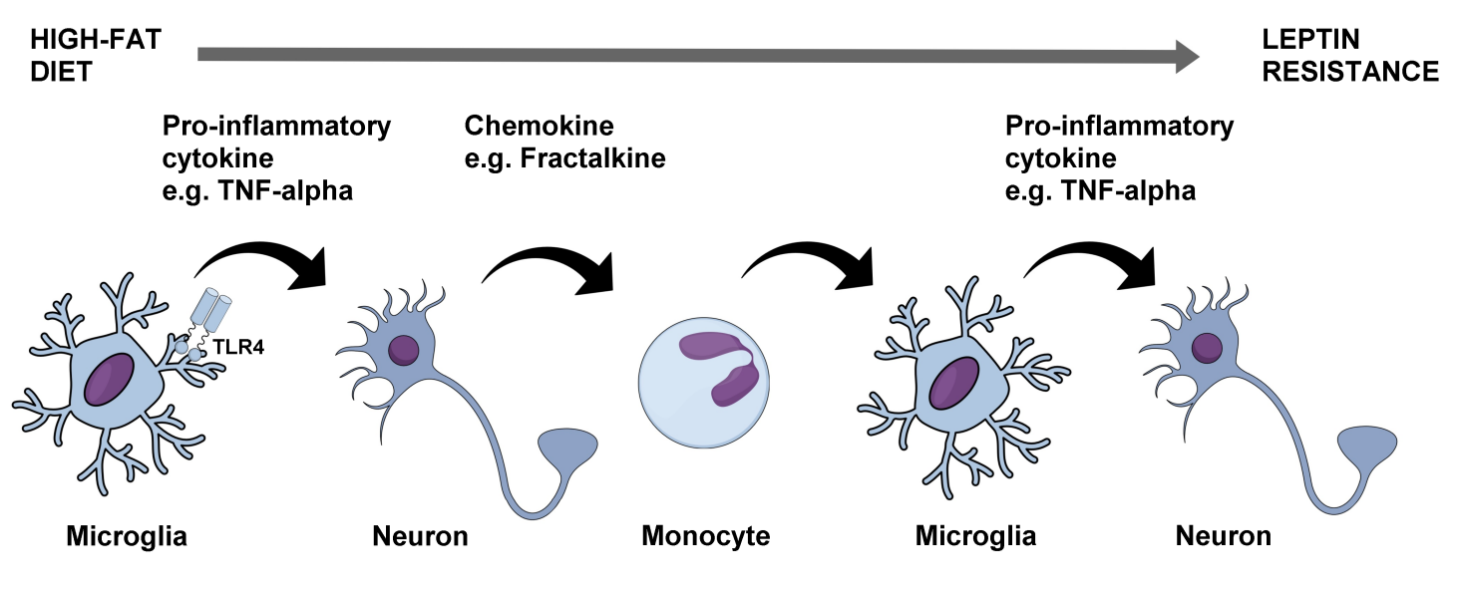
FIGURE 3: Molecular and cellular processes linking chronic high-fat diet consumption with leptin resistance and obesity. The rise in circulating saturated fatty acids from chronic consumption of a high-fat diet leads to activation of TLR4 in hypothalamic microglia which then release pro-inflammatory cytokines such as TNF-α. This in turn leads to the release of the chemokine fractalkine from adjacent neurons which recruits circulating monocytes into the hypothalamus and which then differentiate into activated microglia. A vicious cycle is thus initiated which progressively worsens leptin resistance in hypothalamic neurons contributing to increased food intake and obesity. TLR4 – toll-like receptor 4, TNF-alpha – tumor necrosis factor alpha.

The brain's support and immune cells would then be added to the mix when it was shown that hypothalamic astrocytes and microglia become activated within days after placing rats and mice on a high-fat diet [21]. Subsequent and prior studies suggested that elevated circulating saturated fatty acids themselves act as pro-inflammatory signaling molecules on hypothalamic neurons and microglia through TLR4 [22-24]. In contrast, astrocytes appear to be activated by saturated fatty acids through a bystander effect [22, 24]. Contributions of IKK-β in hypothalamic neurons [12] and microglia [25] to promoting leptin resistance and obesity are now clear. Furthermore, hypothalamic microglial IKK-β signaling promotes the recruitment of circulating CD169+ monocytes into the hypothalamus, which then adopt a microglia-like phenotype to further aggravate inflammation and perpetuate leptin resistance [25]. This, although controversial [26], could be mediated in part through the release of the chemokine fractalkine from hypothalamic neurons consequential to receiving TNF-α from neighboring glial cells [27]. Interestingly, IKK-β signaling in hypothalamic astrocytes seems to serve a different kind of function by shortening their fine processes in the face of a high-fat diet leading to reduced gamma amino butyric acid (GABA) reuptake from the extra-synaptic space [28]. As a result, GABA_B_ receptors are activated in nearby neurons decreasing their production of anorexigenic brain-derived neurotrophic factor (BDNF) which ultimately causes hyperphagia and obesity [28, 29] Thus, the hypothalamic molecular and cellular perturbations in response to chronic high-fat diet consumption are multifaceted, involving a complex array of signaling molecules and cell types originating both peripherally and centrally which act in tandem to disrupt whole-body energy balance regulation (**Figure 3**).

## HYPOTHALAMIC ER STRESS RELIEVERS ARE POTENT WEIGHT LOSS COMPOUNDS

Because of the pivotal role hypothalamic ER stress plays in leptin resistance and obesity development [12, 13, 19, 30], a screen was performed to identify small molecules which might promote weight loss through its amelioration [31]. By comparing the mouse hypothalamic transcriptomic response to obesity and ER stress-relieving chemical chaperones with that of human cell lines treated with a panel of FDA-approved drugs and other bioactive compounds [32], the thunder god vine root extract celastrol emerged as one that caused the most similar absolute changes. Next, in a three week feeding study performed on high-fat dietinduced obese mice, once daily intraperitoneal celastrol injections produced a striking 30% weight loss largely through reduced food intake. This is far greater than the 5-10% typically observed with currently prescribed obesity medications such as the 5 hydroxytryptamine 2C (5-HT2_C_) receptor agonist lorcaserin or the glucagon-like peptide 1 (GLP-1) analogue liraglutide and approaches that found with bariatric surgeries such as vertical sleeve gastrectomy (VSG) and Roux-en-Y gastric bypass (RYGB). That weight loss did not occur in leptin-deficient *ob/ob* or leptin receptor-deficient *db/db* mice treated with celastrol provided strong evidence that it acts as an endogenous leptin sensitizer. Accordingly, hypothalamic leptin receptor signaling was enhanced in wild-type mice after celastrol treatment alongside reduced ER stress although experiments with celastrol administered to mice lacking functional leptin receptors specifically in various nuclei of the hypothalamus [33] still need to be performed to draw definitive conclusions. Celastrol also impressively prevented weight gain in mice placed on a high-fat diet for a year and was well tolerated.

Motivated by this success, the same group of researchers went on to search for compounds which produce a similar gene expression profile as celastrol in mouse embryonic fibroblasts [34]. The winter cherry plant extract withaferin-A emerged as the best hit. Comparable to celastrol, withaferin-A produced approximately 20% weight loss in high-fat diet-induced obese mice in a three week feeding study largely by reducing food intake. Again, withaferin-A was minimally effective in *ob/ob* and *db/db* mice and reinstated the appetite suppressing effects of exogenous leptin treatment in otherwise leptin resistant, high-fat dietinduced obese mice. Finally, as with celastrol, hypothalamic leptin receptor signaling was enhanced and ER stress was reduced by withaferin-A. Notably, precisely how celastrol and withaferin A reverse hypothalamic ER stress in obesity remains unknown. For celastrol at least, this may be from direct inhibition of IKK-β catalytic activity through targeting cysteine 179 in the activation loop [35] and/or non-competitive inhibition of PTP1B [36]. Interestingly, unlike celastrol, withaferin-A does not inhibit PTP1B catalytic activity [36] which may explain why the former is the superi- or weight loss compound. It is also still unclear what effects both these molecules have on hypothalamic glial cells.

In addition to its central mode of action in suppressing energy (food) intake, celastrol has also been proposed to promote a negative energy balance by increasing energy expenditure through stimulating adipose tissue thermogenesis [37]. This is thought to be from the stabilizing effect of celastrol on the protein-protein interaction between heat shock factor 1 (HSF1) and peroxisome proliferator-activated receptor gamma coactivator 1-alpha (PGC- 1α), two transcription factors that induce a thermogenic gene expression program in adipocytes by binding to the *Pgc1*α promoter [37]. Indeed, normal weight mice placed on a high-fat diet for two weeks and treated with low doses of celastrol were protected from weight gain associated with increased energy expenditure but no reductions in food intake [37]. Furthermore, the marked upregulation of thermogenic genes in adipose tissue caused by celastrol was not seen in HSF1-deficient mice. This peripheral mode of action for celastrol was however not supported by subsequent findings from mice deficient in uncoupling protein 1 (UCP1), the principal thermogenic effector in adipose tissue [38]. It is nevertheless still possible that UCP1-independent thermogenesis contributes to celastrol's effects on body weight. These issues notwithstanding, there is genuine hope that natural, safe and effective obesity treatments are in the horizon. However, the effects of both celastrol and withaferin-A need to be evaluated in human individuals with obesity first before metabolic researchers will need to hang up their lab coats.

## VSG RELIEVES HYPOTHALAMIC INFLAMMATION, GLIOSIS, AND ER STRESS

If the pharmacological and surgical forms of obesity treatment described above cause comparable magnitudes of weight loss, then it can be argued that they both have similar mechanisms of action. Indeed, recent studies on rats and mice in the context of obesity-associated infertility and hypertension have shown that VSG reduces hypothalamic inflammation [39, 40], gliosis [39], and ER stress [40]. Interestingly, there is also evidence from mice [41, 42], rats, [43] and humans [44] that like celastrol, VSG induces a thermogenic program in adipocytes although the functional relevance of this remains unclear.

In the study of Xiang *et al*. [39], Sprague Dawley rats were placed either on a standard chow diet or on a high-fat diet. After 16 weeks, half of the high-fat group was randomized to receive VSG whereas the remaining rats received sham surgery to control for the stress of laparotomy. The VSG-operated rats lost approximately 25% of their body weight after eight weeks, which is comparable to that of the human procedure, while the sham-operated groups continued to gain weight during this time period. Immunohistochemical analysis was then performed on hypothalamic sections. Levels of the chemokine monocyte chemoattractant protein 1 (MCP1) were decreased in the VSG-operated group compared to the sham-operated group on a high-fat diet and approached the levels found in the sham-operated group on the low-fat chow diet. Similar findings were made on the levels of pro-inflammatory phosphorylated signal transducer and activator of transcription 3 (pSTAT3) specifically in hypothalamic microglial cells.

In the study of McGavigan *et al*. [40], high-fat dietinduced obese C57BL/6J mice received either VSG or sham surgeries. A subgroup of sham-operated mice was then weight-matched to the VSG group by chronic caloric restriction – an important control to ensure any changes seen are not simply due to weight loss. All groups lost weight during the first two weeks postoperatively, highlighting the sensitivity of mice to surgical stress. However, by the 10^th^ week, VSG-operated mice weighed approximately 10% less than the sham-operated *ad libitum* fed mice, consuming significantly less food during this time period. Hypothalamic lysates were then prepared for Western Blot analysis. Levels of phosphorylated (activated) PERK and eIF2-α were reduced in VSG mice, as was TNF-α. This was not found in the weight-matched control group suggesting that reduced hypothalamic ER stress and inflammation are effects specific to VSG. A selective reduction in *Adlercreutzia* microbiota after VSG was proposed to provide the link between changes in gut anatomy and brain cellular pathology.

**Figure 4:**
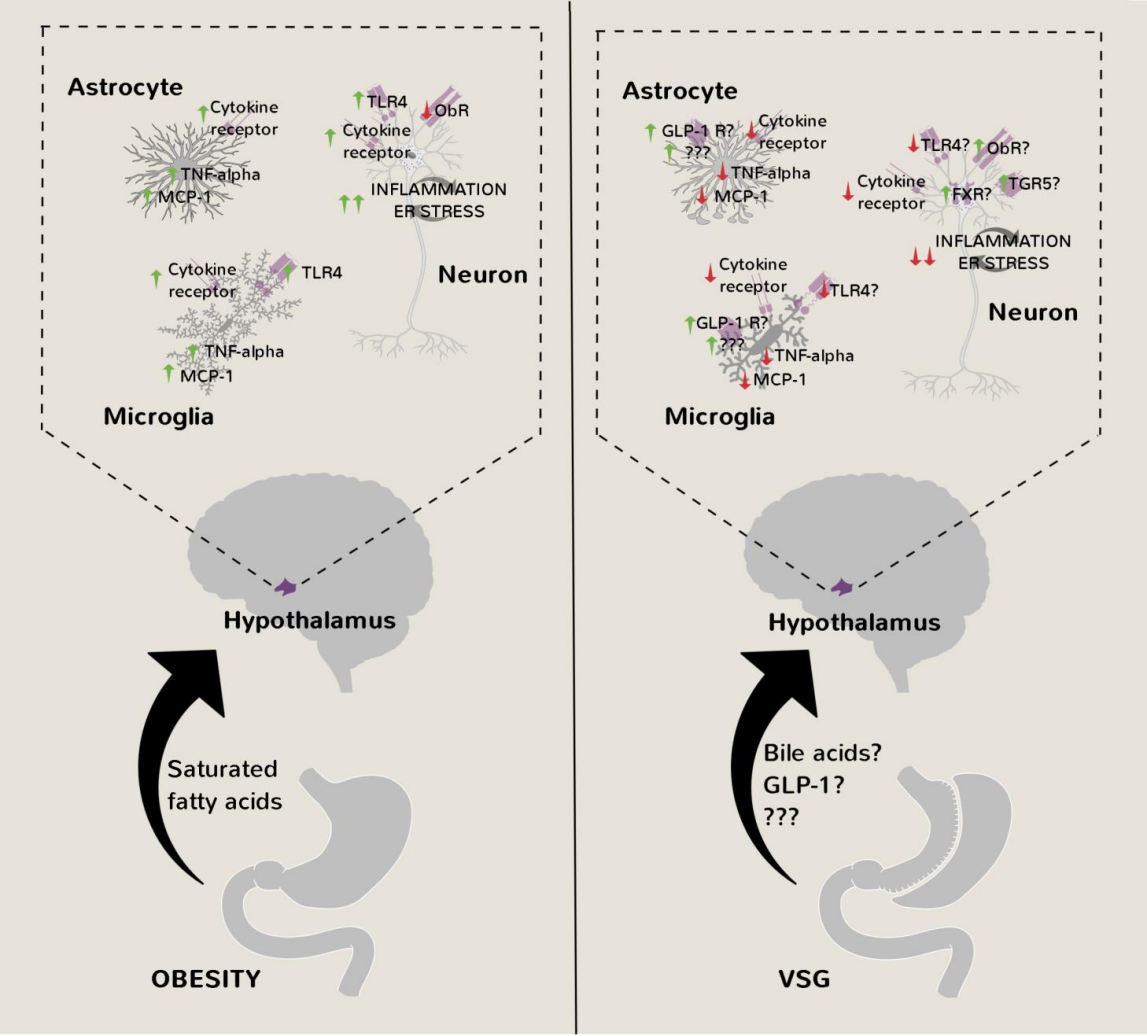
FIGURE 4: Amelioration of hypothalamic inflammation, ER stress and gliosis after VSG. After VSG, activation of FXR/TGR5 in hypothalamic neurons from the rise in circulating bile acids, and GLP-1 receptors in hypothalamic glial cells from the rise in circulating GLP-1, may contribute to amelioration of inflammatory processes, ER stress, gliosis and leptin resistance in obesity, thereby potentially contributing to reduced food intake and lasting weight loss. FXR - farnesoid X receptor, GLP-1 - glucagon-like peptide 1, ObR – leptin receptor, MCP-1 - monocyte chemoattractant protein 1, TGR5 - Takeda G-protein 5 receptor, TLR4 – toll-like receptor 4, TNF-alpha – tumor necrosis factor alpha, VSG - vertical sleeve gastrectomy.

The studies of Xiang *et al*. and *McGavigan* et al., despite only being associational in nature, collectively provide persuasive evidence that VSG reduces hypothalamic inflammation, gliosis and/or ER stress in obesity (**Figure 4**). By extension, it can be reasonably inferred that this particular bariatric surgical procedure restores leptin sensitivity to cause marked and lasting weight loss. In support of this idea, VSG-operated rats and mice have lower circulating leptin levels compared to pair-fed controls with similar adiposity indicative of decreased leptin resistance [45], [46]. Furthermore, VSG-operated rats are more responsive to the acute appetite suppressing effects of exogenous leptin treatment than pair-fed rats [45]. On the other hand, unlike celastrol [24] or withaferin-A [26] treatments, leptin receptor-deficient *fa/fa* Zucker rats [47] and *db/db* mice [48] still lose weight after VSG, suggesting that leptin is a dispensable/redundant mediator of its effects on whole-body energy balance. One potential way to resolve these inconsistencies is to induce hypothalamic ER stress and leptin resistance post-abdominal surgeries in rodents through intracerebroventricular administration of tunicamycin and to assess changes in feeding and body weight [13].

Further questions still remain of course such as the definitive nature of the anti-inflammatory/ER stress relieving gut-derived factor(s) enhanced after VSG. Bile acids acting on hypothalamic farnesoid X receptors (FXR) and/or Takeda G-protein 5 receptors (TGR5) [49-52] are possible candidates as they are essential for the reduced food intake and body weight postoperatively [42, 53, 54]. In this context, bile acids would conceivably be mediating their effects through FXR and TGR5 in hypothalamic neurons and not glial cells [52, 55]. On the other hand, enhanced GLP-1 receptor signaling in hypothalamic astrocytes [56] and/or microglia [57] could explain their reduced activation after VSG thereby contributing to weight loss but again this is not supported by studies on germline GLP-1 receptor deficient mice [58]. Nevertheless, post-embryonic hypothalamic microglial/astrocytic ablation approaches [25, 28, 29] may yield different findings.

## CONCLUSIONS AND FUTURE DIRECTIONS

The fact that chemical hypothalamic ER stress relievers and VSG both can reverse a pathologic brain state in animal models of obesity suggests that rather than just treating its symptoms, they tackle it at one of its root causes. Future work will be required to verify if the promising animal findings can be translated to humans. For example, assessing human hypothalamic gliosis through the use of T2-weighted magnetic resonance imaging [59] or more directly with positron emission tomography [60] may reveal if VSG has an inhibitory effect. Furthermore, patients with higher levels of hypothalamic gliosis may respond better to VSG which would take us one step closer to personalized treatment options for individuals with severe obesity.

## Abbreviations:

eIF2a: elongation initiation factor 2 alpha,
ER: endoplasmic reticulum,
FXR: farnesoid X receptor,
GABA: gamma amino butyric acid,
GLP-1: glucagon-like peptide 1,
IKK-β: inhibitor of kappa beta kinase beta,
IL: interleukin,
HSF1: heat shock factor 1,
NF-Кβ: necrosis factor kappa beta,
PERK: protein kinase R (PKR)-like ER kinase,
PTP1B: protein tyrosine phosphatase 1B,
PGC-1α: peroxisome proliferator-activated receptor gamma coactivator 1-alpha,
SOCS3: suppressor of cytokine signaling 3,
TGR5: Takeda G-protein 5 receptor,
TLR4: toll-like receptor 4,
TNF-α: tumor necrosis factor alpha,
UCP1: uncoupling protein 1,
VSG: vertical sleeve gastrectomy,
vWAT: visceral white adipose tissue.
